# Case Report: Successful Concomitant Pulmonary Thromboendarterectomy and Carotid Endarterectomy

**DOI:** 10.3389/fcvm.2022.839590

**Published:** 2022-05-25

**Authors:** Zhan Liu, Xiaopeng Liu, Xia Zheng, Fan Lin, Guang Sun, Zhidong Ye, Yanan Zhen, Peng Liu

**Affiliations:** ^1^Peking University China-Japan Friendship School of Clinical Medicine, Beijing, China; ^2^Department of Cardiovascular Surgery, China-Japan Friendship Hospital, Beijing, China

**Keywords:** pulmonary thromboendarterectomy (PTE), carotid endarterectomy (CEA), chronic thromboembolic pulmonary hypertension (CTEPH), cerebral protection, carotid stenosis

## Abstract

Pulmonary thromboendarterectomy is the treatment of choice for chronic thromboembolic pulmonary hypertension. Pulmonary thromboendarterectomy concomitant with additional cardiac procedures was reported as safe and feasible. However, the treatment strategy for chronic thromboembolic pulmonary hypertension patients with severe carotid stenosis is still not clear. We describe a case of successful concomitant pulmonary thromboendarterectomy and carotid endarterectomy.

## Introduction

Chronic thromboembolic pulmonary hypertension (CTEPH) is classified as Group 4 pulmonary hypertension (1) and characterized pathologically by organized thromboembolic material and altered vascular remodeling (2). Pulmonary thromboendarterectomy (PTE) is the treatment of choice for CTEPH and potentially curative (3). For CTEPH patients with other cardiac issues, studies have shown that PTE concomitant with additional cardiac procedures, such as defect repair, valvuloplasty, and even heart transplantation, is safe and feasible (4–6). There are some other diseases that may pose a serious threat to patients if left untreated. One of them is carotid artery stenosis. Since PTE surgery is conducted under deep hypothermic circulatory arrest (DHCA), severe carotid artery stenosis can cause irreversible damage to the brain. To date, the treatment strategy for CTEPH patients with severe carotid stenosis is still not clear. We describe a case of a CTEPH patient with severe carotid stenosis who underwent successful concomitant PTE and carotid endarterectomy (CEA).

## Case Description

A 63-year-old male was transferred to our hospital for progressive shortness of breath. Seven months prior to referral, the patient was diagnosed with pulmonary embolism in a local hospital and treated with thrombolysis (alteplase, 100 mg) and anticoagulation (low molecular weight heparin, 100 U/kg, q12h) therapy, and then oral rivaroxaban (10 mg, bid) and bosentan (125 mg, bid) after discharge. However, his clinical condition failed to improve. He was admitted to our hospital and finally diagnosed with CTEPH. The patient did not have any other previous history.

**Figure 1:**
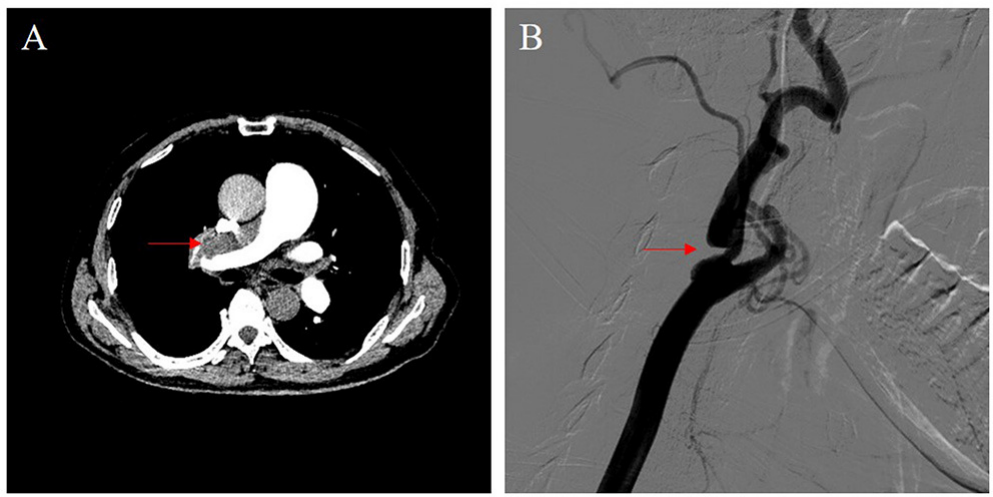
Imaging data. **(A)** Pulmonary computed tomography angiography demonstrated a filling defect located in the main trunk of the right pulmonary artery (red arrow). **(B)** Carotid artery angiography showed severe stenosis of the right internal carotid artery (red arrow).

Transthoracic echocardiography examination showed enlargement of his right heart; the diameter of the right atrium and ventricle was 42 and 48 mm, respectively. Mild to moderate tricuspid regurgitation was detected. Doppler ultrasonography estimated a pulmonary artery systolic pressure of 76 mmHg. The ventilation/perfusion lung scan revealed the presence of mismatched perfusion defects. Pulmonary computed tomography angiography demonstrated filling defects located in the main trunk of the right pulmonary artery and in most lobar and segmental branches of bilateral pulmonary arteries (Figure 1A). Subsequent right heart catheterization (RHC) examination showed severe pulmonary hypertension, with a mean pulmonary pressure (mPAP) of 55 mmHg and pulmonary vascular resistance (PVR) of 1,296 dyn·s·cm^−5^. Moreover, angiography showed severe stenosis of the right internal carotid artery, while the left carotid artery was patent (Figure 1B). An ultrasound showed no evidence of deep venous thrombosis of the lower extremities. The NT-proBNP plasma level was as high as 1,251 pg/mL.

**Figure 2:**
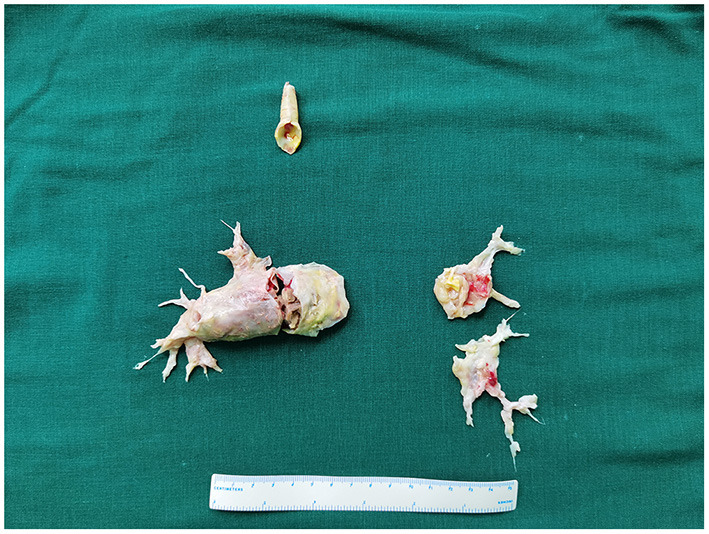
The old thrombus and fibrotic intima were entirely removed.

After discussion by a multidisciplinary team, we decided to perform concomitant procedures of CEA and PTE. We informed the patient and his family of the different treatments and related risks, and they grace approval for concomitant PTE and CEA treatment. After general anesthesia, we first treated the carotid disease with a standard eversion CEA procedure. An oblique longitudinal incision along the anterior edge of the right sternocleidomastoid muscle was made. After systemic heparinization, the common, internal, and external carotid arteries were controlled. The proximal part of the right internal carotid artery was transected, and eversion CEA was performed. The total carotid artery clamp time was 16 min. The internal carotid artery was anastomosed using a 6-0 Prolene suture. Since the PTE procedure was conducted under systemic heparinization and cardiopulmonary bypass, we decided to leave the neck incision opened to prevent hematoma. Pulmonary arteries were exposed through a median sternotomy. Cardiopulmonary bypass was established by ascending aortic and bicaval cannulation. After system cooling to 20 °C, PTE was performed under DHCA. After 3 circulatory arrests, the old thrombus and fibrotic intima were entirely removed (Figure 2). The total circulation arrest time was 57 min, the total CPB time was 315 min, the aortic cross-clamp time was 135 min, and the total operation time was 780 min.

After surgery, mannitol was administered for 3 days and the mean arterial pressure of the patient was controlled at 70–75 mmHg. All these treatment strategies were aimed at preventing cerebral overperfusion and bleeding. The patient recovered uneventfully in the intensive care unit (ICU). The mechanical ventilation time was 113 h, and the ICU length of stay was 6 days. A Swan-Ganz catheter was placed intraoperatively after tracheal intubation was discontinued to measure the postoperative mPAP and PVR. At this time, no narcotics were used and only low-dose phenylephrine (1 μg/kg/min) was administered to control the cardiac index. The postoperative mPAP decreased to 18 mmHg, and the PVR was 200 dyn·s·cm^−5^. The patient was discharged 21 days postoperatively without any complications. During the 5-month follow-up, the patient recovered well without any obvious symptoms. The reviewed RHC examination showed that the mPAP and PVR were 15 mmHg and 136 dyn·s·cm^−5^, respectively. Furthermore, the 6-min walk distance increased from 275 m preoperatively to 435 m postoperatively, and the New York Heart Association functional class improved from grade III to grade I (Table 1). The ultrasound revealed patency of the bilateral carotid arteries. The detailed timeline of the clinical course of this patient is displayed in Table 2.

**Table 1:** The baseline and follow-up parameters of the patient.

**Parameters**	**Baseline**	**Follow-up**
Transthoracic echocardiography		
Diameter of RA (mm)	42	32
Diameter of RV (mm)	48	29
Right heart catheterization		
mPAP (mmHg)	55	15
PVR (dyn·s·cm^−5^)	1,251	136
6-min walk distance (m)	275	435
NYHA functional class	III	I

*RA, right atrium; RV, right ventricle; mPAP, mean pulmonary pressure; PVR, pulmonary vascular resistance; NYHA, New York Heart Association*.

**Table 2:** Detail timeline of clinical course of the patient.

5/4/2018	The patient suffered from progressive shortness of breath.
11/28/2020	The patient was diagnosed with pulmonary embolism in a local hospital and treated with thrombolysis (alteplase, 100 mg) and anticoagulation (low molecular weight heparin, 100 U/kg, q12h) therapy, and then oral rivaroxaban (10 mg, bid) and bosentan (125 mg, bid) after discharge.
6/22/2021	The patient was transferred to our hospital and diagnosed with chronic thromboembolic pulmonary hypertension.
6/25/2021	The preoperative angiography showed severe stenosis of the right internal carotid artery.
6/29/2021	After discussion by a multidisciplinary team, we decided to perform concomitant procedures of carotid endarterectomy (CEA) and PTE.
7/1/2021	Concomitant CEA and PTE was performed successfully.
7/22/2021	The patient was discharged 21 days postoperatively without any complications.
7/22/2021 to present	During the 5-month follow-up, the patient recovered well without any obvious symptoms. Significant improvements were found in hemodynamics, exercise capacity, and functional status during follow-up.

## Discussion

PTE is the treatment of choice for CTEPH and is potentially curative (3). The international registry of incident cases of CTEPH reported a 3-year survival of 90% in those who underwent surgery and 70% in those who did not undergo surgery (7). Clinical guidelines recommend that PTE should be offered to all surgical candidates with CTEPH (2, 8). The evaluation of potential surgical patients mainly includes the evaluation of technical operability and the assessment of the potential risks and benefits of surgery (3). With advances in surgical experience and imaging, PTE could be performed successfully in patients with distal disease (9). On the other hand, the presence of comorbid conditions, with the exception of those that are terminal or end-stage, does not represent an absolute contraindication to PTE (3).

Studies have shown that PTE concomitant with additional cardiac procedures, even heart transplantation, is safe and feasible (4–6). However, the treatment strategy for CTEPH patients with severe carotid stenosis is not clear. Neurological injury is one of the most common perioperative complications of PTE and is associated with cerebral ischemia resulting from DHCA (10). The presence of severe carotid artery stenosis could dramatically increase the risk of perioperative neurological injury. Therefore, carotid stenosis should be resolved before cardiac arrest. After discussion by a multidisciplinary team composed of cardiovascular surgeons, PH physicians, radiologists, anesthesiologists, and perfusionists, we decided to perform concomitant PTE and CEA procedures. One important reason that our team thought concomitant procedures could be feasible was that the postoperative management principles of CEA and PTE are consistent; that is, dehydration and control of cardiac output (or blood pressure). Mannitol (125 mg q12h) was administered, and the cardiac index was controlled at 2.0 to 2.5 L/min/m^2^ after surgery. Therefore, the risk of hyperperfusion after CEA may be lower. The patient recovered uneventfully with significant improvements in symptoms and hemodynamics. During a 5-month follow-up, the patient recovered well without any symptoms of neurological injury. Furthermore, significant improvements were found in hemodynamics, exercise capacity, and functional status during follow-up.

Another option for this patient is staged surgery. To minimize the risk of neurological injury, carotid stenosis should be treated first. Unfortunately, it is difficult for the patient to tolerate CEA surgery under general anesthesia. If pulmonary artery obstruction is left untreated, the patient may not be able to wean from the ventilator after CEA surgery. Furthermore, surgical stimulation may aggravate heart failure and even lead to pulmonary hypertensive crisis. Carotid artery stenting is also feasible for this patient and can be performed under local anesthesia. However, at least 3 months of dual antiplatelet therapy is required after stent implantation, which prolongs the time until PTE surgery and increases the risk of heart failure and death in the patient.

In conclusion, our case demonstrates the first successful concomitant PTE and CEA surgery reported in the literature. For CTEPH patients with severe carotid artery stenosis, concomitant PTE and CEA may be safe and effective. Adequate preoperative evolution by an experienced multidisciplinary team is necessary.

## Data Availability Statement

The raw data supporting the conclusions of this article will be made available by the authors, without undue reservation.

## Ethics Statement

The studies involving human participants were reviewed and approved by the Ethics Committee of the China-Japan Friendship Hospital. The patients/participants provided their written informed consent to participate in this study.

## Author Contributions

ZL and XL wrote the manuscript. XZ, FL, GS, and ZY were involved in data collection and validation. YZ and PL contributed to conceptualization and revised the manuscript. All authors have read and approved the final manuscript.

## Conflict of Interest

The authors declare that the research was conducted in the absence of any commercial or financial relationships that could be construed as a potential conflict of interest.

## Publisher's Note

All claims expressed in this article are solely those of the authors and do not necessarily represent those of their affiliated organizations, or those of the publisher, the editors and the reviewers. Any product that may be evaluated in this article, or claim that may be made by its manufacturer, is not guaranteed or endorsed by the publisher.
